# Redox-related gaseous mediators in the gastrointestinal tract

**DOI:** 10.3164/jcbn.18-56

**Published:** 2018-06-20

**Authors:** Yuji Naito, Kazuhiko Uchiyama, Tomohisa Takagi

**Affiliations:** 1 Molecular Gastroenterology and Hepatology, Graduate School of Medical Science, Kyoto Prefectural University of Medicine, 465 Kajii-cho, Kamigyo-ku, Kyoto 602-8566, Japan; 2 Department of Endoscopy and Ultrasound Medicine, University Hospital, Kyoto Prefectural University of Medicine, 465 Kajii-cho, Kamigyo-ku, Kyoto 602-8566, Japan

**Keywords:** gaseous mediators, gastrointestinal tract, inflammation, microbiome, short-chain fatty acids

## Abstract

Redox-related gaseous molecular species in the gastrointestinal tract are derived from the chemical oxidation-reduction reactions, enzymatic reactions, swallowing, and bacterial production. Recent studies have demonstrated the crucial roles of the microbiota and gaseous molecules in the pathogenesis of gastrointestinal inflammatory and functional diseases. Especially in the hypoxic condition of the large intestine, various bacteria produce acetic acid, methane, and hydrogen sulfide using hydrogen molecules generated by the fermentation reaction as an energy source. In this review, we summarized the recent advances in the biology of redox-related gaseous molecules in the gastrointestinal tract.

## Introduction

**Fig. 1:**
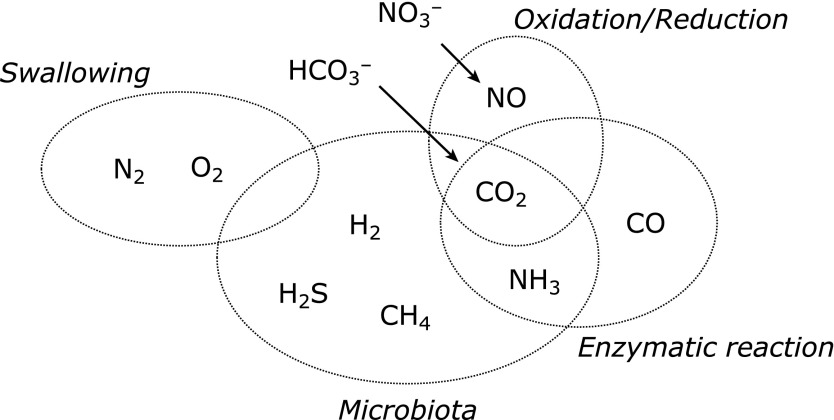
Redox-related gaseous mediators in the gastrointestinal tract.

It is becoming clear that many redox-related gaseous molecular species exist in the gastrointestinal lumen and control the functions of various host cells. Gas species in the gastrointestinal tract are derived from the chemical redox reactions, enzymatic reactions, swallowing, and bacterial production. Nitrogen (N_2_) and oxygen (O_2_) flow into the esophagus and stomach lumen with swallowing. Hydrogen (H_2_), methane (CH_4_), carbon dioxide (CO_2_), and hydrogen sulfide (H_2_S) are produced by the bacteria in the intestinal lumen, ammonia (NH_3_) by urease-positive bacteria, and carbon monoxide (CO) by heme oxygenase (Fig. 1). Although the action of each gaseous molecule on the host and gut microbiota has been reported, there are many unresolved problems concerning the identification of their targets. Recent studies have clarified the importance of the gut microbiota and gas molecules in the pathogenesis of gastrointestinal inflammatory and functional gastrointestinal diseases such as chronic constipation and irritable bowel syndrome, and colorectal cancer. In this review, we introduced the emergence of a new academic field concerning redox-related gaseous molecules in the gastrointestinal tract as well as their association with intestinal diseases and systemic metabolic and degenerative diseases.

## Gut Microbiome and Intestinal Gaseous Molecules

The large intestinal lumen has a very low oxygen concentration and provides a suitable environment for anaerobic bacteria. Short-chain fatty acids such as butyrate are produced by the fermentation inside the lumen using dietary fiber as a substrate, but at the same time, H_2_ gas is produced. Interestingly, gaseous molecules such as acetic acid, CH_4_, and H_2_S are produced using this H_2_ as an energy source, and the gas could affect the physiological function of the host.^(1)^ The gut microbiota plays an important role in the production of these gaseous molecules and interesting findings have been reported along with progress in microbiota analysis. Recent data obtained from gut microbiota in Japanese have indicated characteristic microbiota distanced from the countries of the world. Nishijima and colleagues^(2)^ metagenomically analyzed the human gut microbiome in 12 countries including Japan and found that there are many acetic acid-producing bacteria in Japan, where H_2_ is produced by dietary fiber fermentation. Acetic acid-producing bacteria are relatively characteristic of Japanese people, and H_2_ tends to be used for methane production in 11 other countries including China. Regarding H_2_S production using H_2_, the gut bacteria differs from country to country. The luminal side of the colon can also be said to be an environment filled with diverse gaseous molecules such as H_2_, acetic acid, CH_4_ and H_2_S, involved in redox signals.

It is also a feature of Japanese people that there are few intestinal bacteria that have a repairing function against DNA injury. On the contrary, the intestine in the Japanese may be an environment in which DNA damage is unlikely to occur. It may be characteristic of modern Japanese that the gut microbiota has not been sufficiently adapted to rapid dietary changes such as the increase in high-fat meals. More interestingly, it is also a characteristic of the Japanese that *Bifidobacterium* are predominant. We conducted a 16S rRNA V3–V4 sequence analysis on the gut microbiota of about 300 Japanese people and analyzed the relationship with the Bristol stool consistency scale. Interestingly, both unweighted and weighted UniFrac analysis showed statistically significant differences between male and female.^(3)^ Similar to the previous reports, it was also confirmed that the number of *Bifidobacterium* is relatively high, but 25% of Japanese people have 2% *Bifidobacterium* or less, indicating that the Japanese gut microbiota changes little by little. It has been demonstrated that the outcome of fecal microbiome transplantation in patients with irritable bowel syndrome conducted in Japan is affected by the abundance of this bacteria in donors.^(4)^ A single-arm, open-label study of fecal microbiome transplantation was performed in 10 patients with irritable bowel syndrome. The results showed that *Bifidobacterium*-rich fecal donors may be a positive predictor for successful fecal microbiome transplantation.

## Gut Microbiome and NH_3_

In order to make it possible to live in the gastric acid environment, *Helicobacter pylori* (*H. pylori*) produces NH_3_ by urease using uric acid as a substrate, neutralizing gastric acid locally. Further, monochloramine is produced by the reaction of NH_3_ and hydrogen peroxide/hypochlorous acid via leukocytes recruited to the gastric mucosa after *H. pylori* infection. Monochloramine is more toxic and is involved in mucosal injury and DNA oxidation.^(5)^

In the large intestine, NH_3_ produced by bacteria with urease gene is absorbed and causes hepatic encephalopathy in patients with liver cirrhosis. Recently, it was revealed that NH_3_ produced by intestinal bacterial urease from uric acid is used as a nitrogen source for amino acid synthesis in surrounding bacteria.^(6)^ This NH_3_ metabolism is crucial for the gut microbiota and has attracted attention as one of the interactions between the host and the microbiota. Recent reports have demonstrated that urease-positive bacteria are decreased in inflammatory bowel diseases such as Crohn’s disease, and that dysbiosis of the gut microbiota due to nitrogen deficiency may be involved in the exacerbation of inflammation.^(6)^

## CH_4_ and Intestinal Motility

Information on CH_4_ concentration in humans is relatively limited. CH_4_ concentration varies from 1 to 20 ppm as measured by exhaled CH_4_. It is known that individual variations in CH_4_ concentration and diurnal variation are great. The number of patients suffering from functional intestinal disorders such as chronic constipation and irritable bowel syndrome has increased and this increase became a recent clinical problem. It was suggested that CH_4_ gas is involved in the pathophysiology of these functional diseases. Attaluri *et al.*^(7)^ found that there was a positive correlation between the abundance of methanogenic bacteria and colon transit time. In patients with irritable bowel syndrome with methanogenic bacteria, the post-meal serotonin concentration decreases, resulting in decreasing intestinal motility. Parthasarathy *et al.*^(8)^ compared the microbiota in the feces, mucosa-associated microbiota, colon transit time, and CH_4_ production in patients with chronic constipation with delayed intestinal transit time. As a result, after adjusting for diet and colonic transit, the profile of the mucosa-associated microbiota could be used to distinguish patients with constipation from healthy individuals. The profile of the fecal microbiota was associated with colonic transit and methane production, but not constipation.

## Gut Microbiome and H_2_

Short-chain fatty acids and H_2_ are produced in the course of gut bacterial fermentation using dietary fiber as a substrate. It is also obvious that there are many H_2_-producing bacteria in the intestine of the Japanese. Although the molecular mechanism of H_2_ in the large intestine is unclear, it has recently been reported that the number of H_2_-producing bacteria is significantly smaller in Parkinson’s disease than that in the healthy control as a result of gut microbiome analysis in Japanese patients with Parkinson’s disease.^(9)^ Furthermore, it was also shown that the abundance of the *Bifidobacterium* genus is lower in advanced patients compared with that in the group with stable activity among patients with Parkinson’s disease.^(10)^ Although the relationship between H_2_ and Parkinson’s disease pathology needs to be studied in the future, aggregates of phosphorylated α-synuclein protein, a causative molecule of Parkinson’s disease, have also been detected in the intestinal nerve plexus. The involvement of the gut microbiota in the formation of abnormal protein aggregation in the plexus is beginning to be elucidated.

We reported that acute administration of H_2_ water did not protect against gastric mucosal injury in rats, but chronic administration significantly inhibited gastric injury induced by aspirin, a non-steroidal anti-inflammatory drug.^(11)^ Considering the possibility that chronic administration of H_2_ water may affect the gut microbiota, a four-week chronic administration was performed in mice. As a result, the gut microbiota determined by 16S rRNA metagenomic analysis was clearly distinguished in the principal coordinate analysis in the H_2_ water group as compared with that in the control group, by a significant decrease in Actinobacteria and a significant increase in Deferribacteres at the phylum level.^(12)^ Intestinal environmental research focusing on H_2_ is now ongoing.

## CO as an Anti-Inflammatory Mediator

CO is synthesized from heme oxygenase (HO) in living organisms. The concentration of CO gas in the large intestinal lumen of patients with ulcerative colitis is clearly higher than that in healthy subjects or in the inflammation-healing phase.^(13)^ In immunohistological examinations, inducible HO (HO-1) is positive in macrophages/monocytes of the large intestinal mucosa, and CO may be involved in the activation and differentiation of these cells.^(14,15)^ Even in studies using the experimental colitis model, HO-1 protein and HO-1-positive cells increased in the colonic mucosa of dextran sulfate sodium-induced colitis, and zinc protoporphyrin, an HO-1 inhibitor, significantly aggravated intestinal inflammation.^(16)^ In addition, administration of hemin, an inducer of HO-1, could ameliorate intestinal inflammation.^(17)^ These results indicated that HO-1 is a target molecule for the treatment of intestinal inflammation and that CO is involved in its anti-inflammatory action.

There are new developments concerning the suppression of gastrointestinal inflammation using CO-releasing drugs. We have revealed that CO gas inhalation,^(18)^ CO-releasing molecules (CORMs),^(19)^ and CO-saturated solutions^(20)^ suppressed experimental colitis, and that myofibroblasts, epithelial cells, lymphocytes, and macrophages are involved as target cells of CO gas.^(21)^ Although anti-inflammatory actions have been reported with CORMs using an *in vivo* model, application in humans is difficult because the central metal of the compound is ruthenium. Recently, a tablet containing CORM2 enclosed in a special membrane was newly prepared. The gas is liberated from the tablet because it covers the CORM2 with a special membrane, but ruthenium is discharged as it is in the feces. Orally administering a tablet of approximately 1 mm in size significantly inhibited trinitrobenzenesulfonic acid-induced colitis in mice.^(22)^ Recently, we explored the efficacy and mechanisms of action of CORMs in a T-cell transfer-induced colitis model in mice. The results showed that CORMs conferred protection against the development of intestinal inflammation and attenuated Th17 cell differentiation. Hence, the observed immunomodulatory effects of CORMs could be useful in the development of novel therapeutic approaches for managing intestinal inflammation through the regulation of Th17 differentiation.^(23)^ CO can also act directly on the gut microbiota and is involved in the innate immune response. Bacterial lipopolysaccharide induces HO-1 via toll-like receptors in macrophages, CO produced by HO-1 acts on intestinal bacteria to cause release of adenosine triphosphate (ATP), and ATP activates inflammasome response and interlukin-1β production, indicating that CO is involved in the convergence of inflammation.^(24)^

## Gut Microbiome and H_2_S

Recently, Chassard *et al.*^(25)^ reported the involvement of sulfate-reducing bacteria in constipation-type irritable bowel syndrome (IBS-C). Sulfate-reducing bacteria produces H_2_S using H_2_, and it has been shown that H_2_S affects intestinal motility and visceral nerve hypersensitivity. Compared to the healthy group, sulfate-reducing bacteria are abundant and sulfides production is enhanced compared to butyric acid production in the IBS-C group. Especially, transient receptor potential ankyrin-1 (TRPA1) and T-type Ca^2+^ channels (Cav 3.2), which are nociceptors expressed in sensory nerves are activated by H_2_S or its oxidation product polysulfide, indicating the possible association with visceral hypersensitivity.^(26,27)^ Generally, in order to produce H_2_S, sulfate ions (SO_4_^2−^) in high concentration and organic substances to become a carbon source, sulfate-reducing bacteria are necessary in an anaerobic environment and in the presence of water, which is similar to the large intestinal environment. *Bilophila wadsworthia* using taurine as a substrate, *Fusobacterium nucleatum* using cysteine and *Desulfovibrio bacterium* are known as sulfate-reducing bacteria in humans. *Fusobacterium nucleatum* is frequently detected in colon cancer tissues and in relation to carcinogenesis.^(28)^ *Bilophila wadsworthia* has been found to be associated with high-fat/high-protein diets and produces H_2_S using taurine-conjugated secondary bile acid as a substrate.^(29)^ In either case, although it was demonstrated that sulfate-reducing bacteria are relatively few in the Japanese gut microbiota, it is reported that the increase of this bacteria due to the rapid dietary change might be associated with the increase in patients with chronic constipation or colorectal cancer.

## Conclusion

We introduced numerous redox-related gaseous molecular species in the gastrointestinal lumen that control the function of various host cells. It is also becoming possible to analyze the large amounts of data derived from metagenomic analysis of the gut microbiome, metabolome analysis of the intestinal environment, and host inflammatory/immune response with the help of artificial intelligence. As a result, a novel therapeutic target molecule is being found, especially in the clinical medicine. There are unsolved problems regarding the influence on intracellular signals, especially redox signals, by gaseous molecular species.

## Author Contributions

YN, TT and KU were involved in editing the manuscript. All authors discussed the results and commented on the manuscript.
